# Global Oxygen Detection in Water Using Luminescent Probe on Anodized Aluminum

**DOI:** 10.3390/s90604151

**Published:** 2009-06-02

**Authors:** Hirotaka Sakaue, Tatsuya Ozaki, Hitoshi Ishikawa

**Affiliations:** 1 Aerodynamic Research and Development Directorate, Japan Aerospace Exploration Agency / Chofu, Tokyo 182-8522, Japan; 2 Department of Mechanical Engineering, Tokyo University of Science / Chiyoda, Tokyo 102-0073, Japan; E-Mails: tatsumas@chofu.jaxa.jp (T.O.); ishi@rs.kagu.tus.ac.jp (H.I.)

**Keywords:** global measurement, optical technique, dissolved oxygen, anodized aluminum, pressure-sensitive paint

## Abstract

We have developed anodized-aluminum pressure-sensitive paint (AA-PSP) as a global oxygen sensor in water. Platinum (II) *meso*-tetra(pentafluorophenyl)porphine is selected as a luminophore based on a dipping deposition study. The developed AA-PSP is characterized using water calibration setup by controlling dissolved oxygen concentration. It is shown that AA-PSP yields 4.0% change in luminescence per 1 mg/L of oxygen concentration at 23°C. Other characteristics, such as temperature dependency, photo-degradation, and physical stability, are discussed in this paper. This AA-PSP is used to demonstrate its capability of global oxygen detection in water using the impingement of oxygen rich water (20 mg/L). Even though the difference in water is only the concentration of oxygen, we can obtain global oxygen information of the jet impingement using a fast frame rate camera. Oxygen maps as well as cross-sectional distributions are shown every 0.1 s.

## 1. Introduction

Oxygen detection in water is essential in various fields, such as medical, biochemical, and microbial studies, as well as our living environment, such as agriculture, foods, drugs, natural waters, and sewage wastes [1]. There are commercially available electrodes and optrodes for oxygen detection. Electrodes use an electrochemical method to determine the oxygen concentration [2], while optrodes use luminescence lifetimes or intensities related to the oxygen concentration. The former have limitations in that they consume oxygen in water, thus varying the local oxygen concentration around the detecting probe. Campo *et al.* reported the latter method to detect dissolved oxygen in water [3]. In their method, an Al-Ferron oxygen detecting probe in a sol-gel is covered with a nylon membrane that reduced its time response to the oxygen change on the order of minutes. Commercially available detectors using electrodes and optrodes give point wise measurement that may limit their measurement applications.

**Figure 1.**
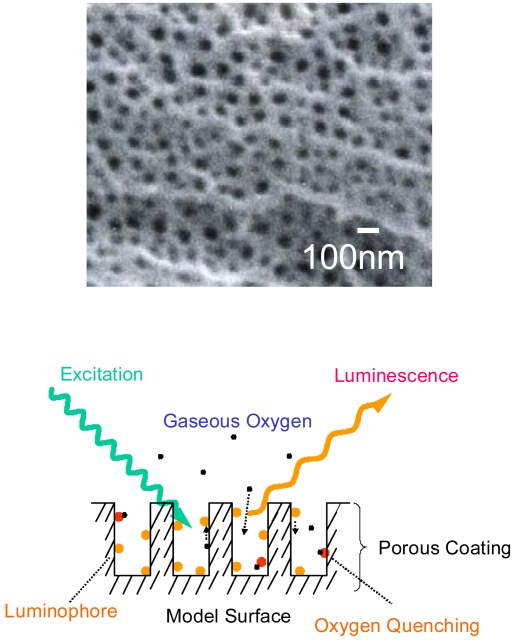
(a) Nano-open structure of anodized aluminum surface. Surface image was taken using a scanning electron microscope. (b) Schematic description of anodized aluminum pressure sensitive paint (AA-PSP).

In aerospace engineering, anodized-aluminum pressure-sensitive paint (AA-PSP) has been used in wind tunnel measurements [4]. Because of its nano-open structure [Figures 1 (a) and (b)], AA-PSP yields high mass diffusion that results in its pressure response time on the order of 10 μs [5]. This structure also gives advantages that it is sensitive to low mole fraction of oxygen on the order of a few ppm [6]. By applying AA-PSP, we can obtain global surface oxygen information instead of point wise information that may result in wide applications in oxygen detection fields. Due to its open structure, AA-PSP may give fast time responses in water as well.

In this paper, we present the development of AA-PSP as a water sensor for global oxygen detection. We characterized this sensor in terms of oxygen sensitivity, temperature dependency, photo-degradation, and physical stability. A demonstration of global oxygen detection in water using our developed AA-PSP is also included.

## 2. Characterization

### 2.1. Dipping Deposition and Luminophore Determination

**Figure 2.**
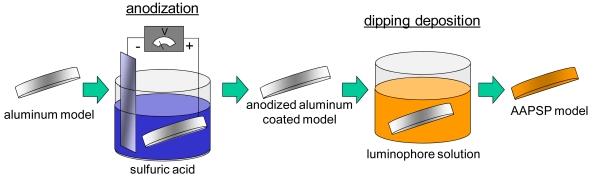
Schematic description of dipping deposition method.

**Figure 3.**
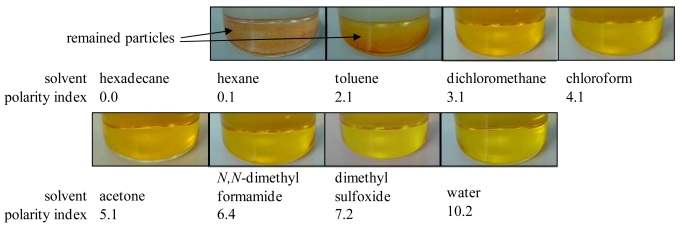
Pictures of various luminophore solutions with bathophen ruthenium as a luminophore. Eight solvents were selected based on their polarity index.

A luminophore as an oxygen probe was applied on an anodized aluminum surface by the dipping deposition method [7]. This method requires a luminophore, a solvent, and an anodized aluminum coating. The application procedure is schematically shown in Figure 2. The luminophore was dissolved in solvents which were varied according to their polarity index. Depending on the polarity index of the solvents, the luminophore changed its dissolution status (Figure 3). The luminophore used shown in Figure 3 was bathophen ruthenium, which is a commonly used luminophore for AA-PSP. Solvents of polarity index greater than 3.1 dissolved bathophen ruthenium, while solvents with lower polarity index did not dissolve the luminophore. The anodized aluminum coating was dipped in these solutions or mixtures. We could coat the luminophore with some of these solutions. For the case of bathophen ruthenium, dichloromethane applied the luminophore well on the anodized coating [7]. Based on this solvent study, we can apply luminophore on the anodized surfaces.

**Figure 4.**
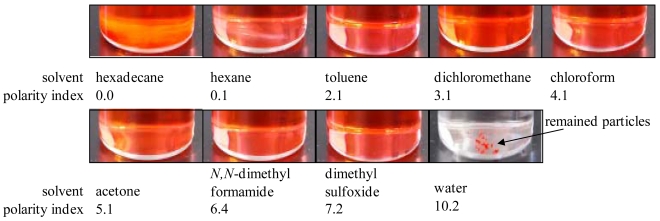
Pictures of solvent study with PtTFPP as a luminophore. Nine different solvents were selected by their polarity index.

**Figure 5.**
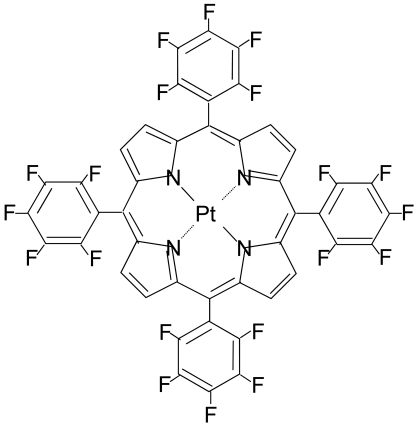
Chemical structure of PtTFPP. F: fluorine, Pt: platinum, and N: nitrogen, respectively.

To develop AA-PSP as a water sensor, the luminophore should not dissolve in water, because the porous surface, where the luminophore is applied, is open to water environment. One can see in Figure 3 that the bathophen ruthenium luminophore was dissolved in water so that this luminophore was not suitable for our water sensor. Platinum (II) *meso*-tetra(pentafluorophenyl) porphine (PtTFPP) from Frontier Scientific is another candidate as a luminophore. Figure 4 shows the solvent study of PtTFPP. One can see that PtTFPP was not dissolved in water. This luminophore is protected by fluorine, which indicates that it is water repellant (Figure 5). Based on the solvent study, PtTFPP was applied on the anodized aluminum surface in hexadecane as a solvent. In Table 1, we summarized the conditions of dipping deposition method to prepare AA-PSP as a water sensor. Anodized aluminum coating was prepared following Sakaue's procedure [7]. The coating thickness was 10 μm ± 1 μm measured with an eddy current apparatus (Kett, LZ-330).

### 2.2. Oxygen Calibration in Water

**Figure 6.**
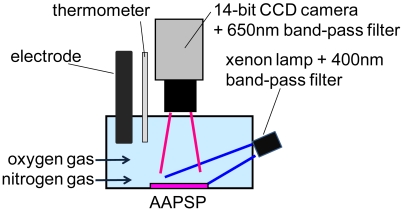
Schematic description of oxygen calibration setup in water.

Figure 6 shows our oxygen calibration setup. The developed AA-PSP was placed at the bottom of a water tank. It was illuminated by a 300 W xenon lamp (Hamamatsu Photonics) through a 400 ± 50 nm band-pass filter. The illumination output from the lamp was guided through an optical fiber. The fiber exit was located 90 mm from the AA-PSP. The luminescent image was acquired by a 14-bit CCD camera (Hamamatsu Photonics, C4742-98-24EW) through 650 ± 50 nm band-pass filter to exclude the illumination wavelength. The camera exposure was 2 s. Oxygen concentration in water was adjusted by oxygen and nitrogen gas injection. The concentration was monitored by a conventional electrode sensor (Fuso Rikaseihin, DO-5509). Distilled water was used and its temperature was kept constant at 23 °C.

**Figure 7.**
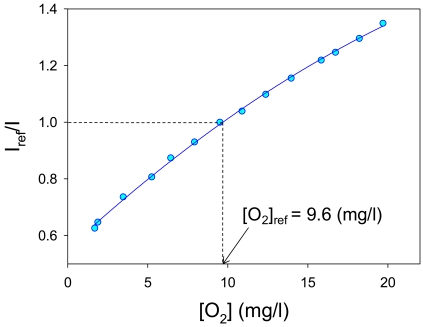
Oxygen calibration result.

Based on the Stern-Volmer principle, oxygen quenching of AA-PSP can be described as the following equation [8]:

(1) $$\frac{I_{0}}{I}=1+K_{SV}[O_{2}]$$

where *I* is luminescent intensity, *K_SV_* is the Stern-Volmer quenching constant, [*O_2_*] is dissolved oxygen in water, and the subscript *0* denotes the conditions without oxygen, respectively. AA-PSP measurement uses a reference image, *I_ref_*, to remove a non-uniform illumination and PSP non-uniformity:

(2) $$\frac{I_{0}}{I_{\text{ref}}}=1+K_{SV}{\left[O_{2}\right]}_{\text{ref}},$$

where the subscript *ref* denotes the reference conditions. The equation (1) can be described using *I_ref_* in equation (2). Dividing equation (1) by equation (2) gives the following relationship:

(3) $$\frac{I_{\text{ref}}}{I}=A+B[O_{2}],$$

where *A* and *B* are calibration constants. Unfortunately, AA-PSP has a non-linear relationship with [*O_2_*] due to the surface adsorption on the anodized aluminum coating [7]. To provide a better calibration fit to convert the luminescent image to oxygen concentration, equation (3) is described in a polynomial form. In our present study, a second ordered polynomial form was used, that is:

(4) $$\frac{I_{\text{ref}}}{I}=c_{0}+c_{1}[O_{2}]+c_{2}{\left[O_{2}\right]}^{2},$$

where *c_0_, c_1_*, and *c_2_* are calibration constants, respectively. Figure 7 shows an oxygen calibration result fitted with equation (4). We used a standard oxygen concentration of 9.6 mg/L in water at 23 °C as a reference.

Oxygen sensitivity (%/mg/L) is defined as the slope of calibration at reference. From the equation (4), oxygen sensitivity is described as the following. The oxygen sensitivity of our developed AA-PSP is 4.0 %/mg/L calculated using:

(5) $${\frac{d\left(I_{\text{ref}}/I\right)}{d[O_{2}]}|}_{[O_{2}]={\left[O_{2}\right]}_{\text{ref}}}=c_{1}+2c_{2}{\left[O_{2}\right]}_{\text{ref}}.$$

**Figure 8.**
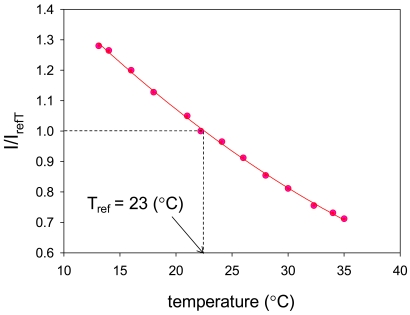
Temperature calibration result.

AA-PSP as well as PSP in general has a temperature dependency [9]. This influences the luminescent signal. We plotted the luminescent signal related to temperature (Figure 8). The plot was fitted with the second order polynomial, described in equation (6). We selected the reference luminescent signal, *I_refT_*, at 23 °C with constant oxygen concentration at 9.6 mg/L. We have:

(6) $$\frac{I}{I_{\text{refT}}}=c_{T0}+c_{T1}T+c_{T2}T^{2},$$

where *c_T0_, c_T1_*, and *c_T2_* are calibration constants, respectively. This calibration plot describes the decrease in luminescent signal with increasing temperature. The temperature dependency is defined as the slope of the temperature calibration at reference. The temperature dependency was found to be -2.8 %/°C using:

(7) $${\frac{d\left(I_{\text{refT}}/I\right)}{dT}|}_{T=T_{\text{ref}}}=c_{T1}+2c_{T2}T_{\text{ref}}.$$

**Figure 9.**
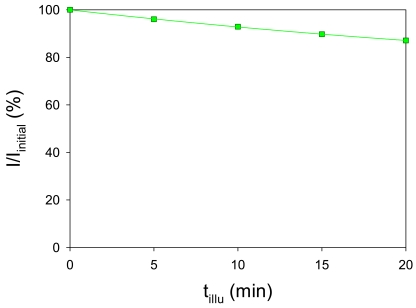
Photodegradation of AA-PSP in water.

The luminophore experiences photo-degradation under illumination due to its photo-physical process [9]. Our developed AA-PSP was illuminated continuously to evaluate its photo-degradation. We used the same calibration setup described in Figure 6 with constant oxygen concentration of 9.6 mg/L at 23 °C. Figure 9 shows a photo-degradation of our developed AA-PSP. The initial luminescent signal, *I_initial_*, was set at 100%. As the illumination time, *t_illu_*, increased, the luminescent signal strength decreased by 13% in 20 min. We fitted with a second order polynomial to determine the rate of degradation (%/min), which is -0.8 %/min at the initial time.

### 2.3. Discussion: Stability Test

**Figure 10.**
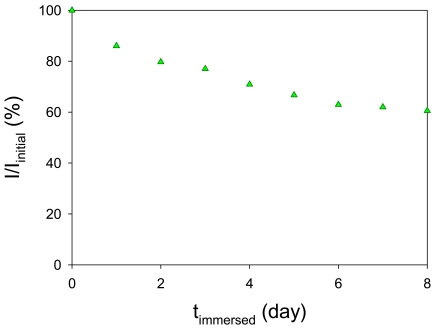
Luminescent signal change as AA-PSP immersed in water for eight days.

We immersed our AA-PSP in the water tank for eight days to study its physical stability. The initial luminescent signal, *I_initial_*, was set at 100 % at a constant oxygen concentration of 9.6 mg/L at 23 °C. Figure 10 shows the luminescent signal change over this period, where one can see the decrease in the signal. After eight days, the signal had decreased by 39.4 %. The magnitude of the signal decrease was large up to around four days, but the amount then became small as the days passed. Once the number of immersed days increased to sixteen, the signal became fairly stable. One of the factors in the decrease in the signal is the photodegradation of the luminophore. Even though the camera exposure was 2 s, we illuminated longer than this period including opening and closing the illumination shutter. These extra times amount to roughly 4 s for each image acquisition. There were in total 293 data points for all characterizations from the initial day to the eighth day that include oxygen calibrations, temperature calibrations, and stability test. The overall illumination period was about 1,758 s. Based on the photodegradation test, this gives a 23.4 % of decrease in luminescent signal. However, this amount is not enough to explain the overall decrease in luminescent intensity over eight days. Another factor may be scale from water. The water in the tank was kept constant without any filtering. We noticed that the AA-PSP developed a thin layer of scale on the surface after eight days in water. We think that this may reduce the excitation and emission of AA-PSP so as to decrease the luminescent signal. We could not detect illumination from water itself that could reveal if the luminophore was not dissolved in water. The signal decrease due to the dissolution of PtTFPP in water is, thus, difficult to identify any other factors.

Oxygen and temperature calibrations were obtained at the initial day as well as over eight days after AA-PSP immersed in the water tank. Table 2 shows the oxygen sensitivities as well as temperature dependencies. Even though the luminescent intensity was changed by 39.4 %, both oxygen sensitivity and temperature dependency variations were relatively smaller with changes as 17.5 % and 11.1 %, respectively.

## 3. Global Oxygen Detection in Water

**Figure 11.**
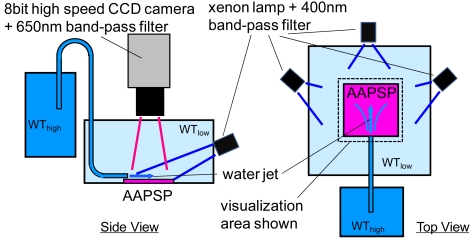
Side and top views of oxygen water impingement setup.

Figure 11 shows side and top views of global oxygen detection setup. AA-PSP, the illumination source, and optical filters were the same as those used in the previous section. We used three illumination sources as well as optical filters in the previous section, which increase the luminescent signal from the AA-PSP. To acquire images with a fast frame rate, an 8-bit high-speed CCD camera (Phantom v4.2) was used. By the illumination and camera setup, the camera exposure could reduce to 9.9 ms. The camera acquired luminescent image at 100 frames per second (100 Hz). We placed the AA-PSP in the water tank, WT_low_, with its oxygen concentration of 3 mg/L. Another water tank, WT_high_, with higher oxygen concentration of 20 mg/L was connected to WT_low_ with a tube. The tube exit was of elliptical shape with its major axis equals 2 mm. The tube exit was placed next to the AA-PSP edge. The water jet from WT_high_ was injected into WT_low_ over the AA-PSP surface. An averaged flow rate was 2.1 g/s. Water temperature was kept constant at 23 °C.

**Figure 12.**
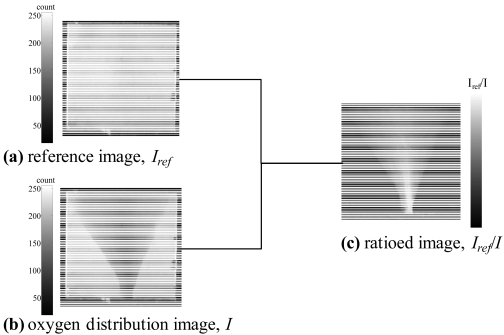
(a) Reference image, *I_ref_*, (b) representative oxygen distribution image, *I*, and (c) ratioed image, *I_ref_/I*.

Figures 12(a) and (b) show the reference image and a representative oxygen distribution image, respectively. The difference in water is only the amount of oxygen. We could not see the difference by our eyes, but AA-PSP did respond to the change of oxygen. We can see the luminescent decrease due to oxygen water impingement that shows in dark gray region in Figure 12(b). With the ratio *I_ref_/I*, we can remove a non-uniform illumination as well as a small AA-PSP non-uniformity. This is shown in Figure 12(c). Based on the oxygen calibration result discussed in the previous section, the luminescent ratio images, *I_ref_/I*, were then converted to oxygen concentration, [*O_2_*] in mg/L.

**Figure 13.**
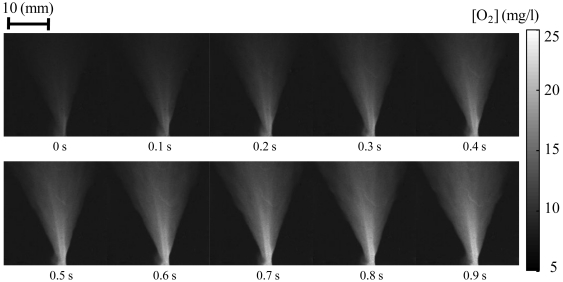
Global oxygen detection results. Oxygen distributions every 0.1 s are shown.

Even though every 100 Hz image (0.01 s) was obtained, the change in jet formation appeared to be relatively small. We chose the oxygen distribution images every 0.1 s to see relatively larger change, which are shown in Figure 13. We chose the initial time at the moment when the jet was beginning to form. One can see the jet formation from the oxygen distribution. The oxygen concentration can be seen from the scale shown in the figure. Because this is a luminescent image technique, we can increase the spatial resolution by using an optical microscope. If the signal level is strong enough for image acquisition system, the absolute limitation of the spatial resolution may be the size of luminescent molecules.

**Figure 14.**
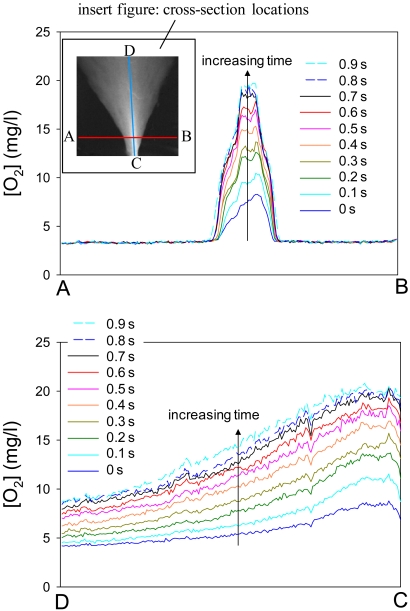
Oxygen distributions at cross-sections A–B and C–D. The insert figure shows locations of A – B and C – D. Distributions are shown every 0.1 s.

Because the results were obtained as images, we can choose an arbitrary point or line to monitor the oxygen concentration variation. Figures 14(a) and (b) show oxygen distribution varying with time at a cross-section A–B and C–D, respectively. The distributions are shown every 0.1 s. These results show that by using AA-PSP as a global oxygen sensor, we can visualize and detect global oxygen distribution in water.

### 3.1. Discussion: AA-PSP response in water

At present, there is no instrument available to evaluate the response time of AA-PSP in water experimentally. However, we roughly estimated the order of response time based on Kameda *et al.* [5]. They derived the response time of AA-PSP in air using the effective diffusion coefficients. The characteristic response time, *t_0.99_*, is used, which reaches 99 % of the total response to a step change. We use their derivation as an analogy to our case. Assume the effective diffusion coefficient has the same order as that of bulk diffusion coefficient of oxygen in water, *D*, and a step change of oxygen concentration occurred over an AA-PSP surface. The order of *t_0.99_* is:

(8) $$O\left(t_{0.99}\right)=O\left(\frac{h^{2}}{D}\right),$$

where *h* is the thickness of AA-PSP, which has the order of 10^-5^ m, and *D* has the order of 10^-7^ m^2^/s [10]. Then, our estimated response time of AA-PSP in water is on the order of milliseconds.

## 4. Conclusions

Anodized-aluminum pressure-sensitive paint (AA-PSP) was developed as a global oxygen sensor in water. Platinum (II) *meso*-tetra(pentafluorophenyl) porphine was used as a luminophore based on dipping deposition study in water. Calibrations of the developed AA-PSP showed an oxygen sensitivity of 4.0 %/mg/L, a temperature dependency of -2.8 %/°C, and a photo-degradation rate of -0.8 %/min. Stability tests showed that the luminescent signal strength changed by 39.4% after eight days when AA-PSP was immersed in water. The developed AA-PSP was used to demonstrate its capability of global oxygen detection in water using the impingement of oxygen rich water (20 mg/L) into oxygen less rich water (3 mg/L). We showed global oxygen information of the jet impingement as well as cross-sectional distributions in every 0.1 s.

## Figures and Tables

**Table 1.** Conditions of dipping deposition to prepare an AA-PSP as a global water sensor.

**Dipping deposition parameters**	**Conditions**
dipping solution	1 mM of PtTFPP in hexadecane
dipping duration	60 min at 23 °C

**Table 2.** Change in oxygen sensitivity and temperature dependency after eight days.

	**Oxygen sensitivity (%/mg/L)**	**Temperature dependency (%/°C)**
**At start of experiment**	4.0	-2.7
**Eight days later**	3.3	-2.4
